# Supplementary material for: Resolving the full spectrum of human genome variation using Linked-Reads
Source: Genome Res. 2019 Apr;29(4):635–45. doi: 10.1101/gr.234443.118 (PMC6442396; doi:10.1101/gr.234443.118)
Supplement: Supplemental Material [file supp_gr.234443.118_Supplemental_longranger-2.2.zip › longranger-2.2.tar.bz2!/lr2.2-public/lib/stan/cmdstan/stan/doc/api/html/row_8hpp_source.html]

Stan: src/stan/math/matrix/row.hpp Source File


|  |  |
| --- | --- |
|  | Stan  2.5.0  probability, sampling & optimization |


- Main Page
- Related Pages
- Namespaces
- Classes
- Files

- File List
- File Members

All Classes Namespaces Files Functions Variables Typedefs Enumerations Enumerator Friends Macros Pages

- src
- stan
- math
- matrix

row.hpp

Go to the documentation of this file.

1 #ifndef STAN\_\_MATH\_\_MATRIX\_\_ROW\_HPP

2 #define STAN\_\_MATH\_\_MATRIX\_\_ROW\_HPP

3

4 #include <stan/math/matrix/Eigen.hpp>

5 #include <stan/math/error\_handling/check\_greater\_or\_equal.hpp>

6 #include <stan/math/error\_handling/check\_less\_or\_equal.hpp>

7

8 namespace stan {

9  namespace math {

10

23  template <typename T>

24  inline

25  Eigen::Matrix<T,1,Eigen::Dynamic>

26  row(const Eigen::Matrix<T,Eigen::Dynamic,Eigen::Dynamic>& m,

27  size\_t i) {

28  stan::math::check\_greater\_or\_equal("row(%1%)",i,1U,"i",(double\*)0);

29  stan::math::check\_less\_or\_equal("row(%1%)",i,static\_cast<size\_t>(m.rows()),"i",(double\*)0);

30  return m.row(i - 1);

31  }

32

33  }

34 }

35 #endif

stan::math::row

Eigen::Matrix< T, 1, Eigen::Dynamic > row(const Eigen::Matrix< T, Eigen::Dynamic, Eigen::Dynamic > &m, size\_t i)

Return the specified row of the specified matrix, using start-at-1 indexing.

**Definition:** row.hpp:26

check\_less\_or\_equal.hpp

Eigen.hpp

stan::math::check\_less\_or\_equal

bool check\_less\_or\_equal(const char \*function, const T\_y &y, const T\_high &high, const char \*name, T\_result \*result)

**Definition:** check\_less\_or\_equal.hpp:52

check\_greater\_or\_equal.hpp

stan::math::check\_greater\_or\_equal

bool check\_greater\_or\_equal(const char \*function, const T\_y &y, const T\_low &low, const char \*name, T\_result \*result)

**Definition:** check\_greater\_or\_equal.hpp:58


---

[ Stan Home Page ]

*© 2011–2014,
Stan Development Team.*
